# Digital-Twin-Oriented Virtual Training Environment for Agricultural Robot Navigation: A Vineyard Rover Case Study

**DOI:** 10.3390/s26123766

**Published:** 2026-06-12

**Authors:** Gábor Kusper, Zoltán Barócsi, Péter Csóka, Krisztián Vajda, József Sütő

**Affiliations:** 1 Department of IT, Eszterházy Károly Catholic University, 3300 Eger, Hungary; kusper.gabor@uni-eszterhazy.hu; 2 Institute of Viticulture and Enology, Eszterházy Károly Catholic University, 3300 Eger, Hungary; barocsi.zoltan@uni-eszterhazy.hu; 3 InnovITech Ltd., 1037 Budapest, Hungary; peter.csoka@innovitech.hu (P.C.); krisztian.vajda@innovitech.hu (K.V.); 4 Department of IT Systems and Networks, University of Debrecen, 4028 Debrecen, Hungary

**Keywords:** digital twin, virtual training, route planning, agricultural robots

## Abstract

A virtual training environment offers clear advantages for agricultural robotics. It provides a safe setting in which perception, navigation, and control algorithms can be evaluated without risking damage to either the robot or the crop. It also supports efficient data generation: large volumes of training data can be collected under diverse environmental conditions that would be costly, slow, and often season-dependent in real-world deployments. This broader variability improves model adaptability, reduces the risk of overfitting, and leads to more robust operation. In this paper, we argue that digital twin technology should therefore be understood not merely as a passive mirror of a physical robot, but as an active training environment in which multiple sensor-related subprocesses can be developed, tested, validated, and refined jointly. This paper is based on our experiences with digital twin technology used in the development of a vineyard robot, including a self-driving rover, sensor simulation, procedural map generation, and agriculture-specific movement models. Our contribution is threefold: we reinterpret the digital twin as a training space, propose a layered framework for training agricultural robots in virtual environments, and explain why agriculture is a particularly strong use case, given variable field conditions, expensive real-world experimentation, and persistent labor scarcity. To validate this framework, we present the simulation-based evaluation of an autonomous reinforcement learning agent. The agent has been trained entirely in this virtual environment, which successfully navigated to 155 out of 161 target points in a simulated vineyard demonstration environment.

## 1. Introduction

Orchards require numerous operations each year that involve intensive physical labor. In viticulture, pruning is one of the most labor-demanding tasks. Its aim is to ensure the growth of new plant nodes following the winter season. Even today, the vast majority of fruit growers worldwide continue to perform pruning manually [1]. In the case of grape production, the cost of manual pruning is significantly higher than in many other agricultural sectors. Pruning alone may account for as much as 20–30% of the total annual cultivation costs, where mechanization can result in substantial savings above a certain plantation size [2,3].

The global viticulture industry is currently navigating a dual crisis: a chronic shortage of skilled seasonal labor and an escalating need for precision management to mitigate the effects of climate change. Dormant pruning remains the most critical intervention in the vineyard, as it directly regulates the source-sink relationship and determines the vine’s vegetative-reproductive balance for the following season. While traditional mechanical hedging offers operational speed, it lacks the selective precision required to maintain vine longevity and prevent the spread of grapevine trunk diseases [4].

Automated robotic solutions provide increasing opportunities to replace human labor across multiple sectors, including agriculture. Since pruning involves a sequence of repetitive operations, its automation has attracted considerable interest among growers. Modern robotic research aims to automate “respectful” pruning protocols that emphasize maintaining lymphatic sap flow and minimizing large wounding [5]. Reviews of agricultural robotics describe labor shortage as one of the main drivers of robotization [6,7,8]. Vineyard-specific studies also show that skilled pruning work remains difficult for staff to do reliably [9]. However, replicating this expert human judgment requires a seamless integration of high-level viticultural logic with low-level robotic precision within an unstructured environment [10].

The primary focus of this study is autonomous pruning robots, where the main technical bottleneck in achieving autonomy lies in perception and navigation [11]. From a navigation perspective, one of the key challenges is real-time path planning, which also includes obstacle avoidance. Due to the complexity of real-world field conditions and the requirement for precise navigation, this problem cannot be reduced to simple coordinate-based motion [12].

Since the window for physical field testing is limited and hardware-intensive, high-fidelity simulation has emerged as the cornerstone of contemporary development. Although robots are increasingly equipped with a greater number of sensors in order to expand both the quantity and heterogeneity of available data, achieving precise autonomous operation remains a significant challenge. Several review papers published in 2025 also highlight the difficulties associated with autonomous operation [13,14].

At this point, digital-twin-oriented virtual training environments have become highly relevant. In the broader literature, digital twins are discussed as digital counterparts of physical systems that support monitoring, prediction, and reasoning [15,16]. During the development of a pruning robot, significant benefits can be achieved using digital-twin-oriented virtual environments that integrate automation with sensor-based environmental analysis, thereby supporting safer and more systematic development. Within a virtual environment, cyclic tasks such as navigation between grapevine rows can be executed millions of times, thereby enabling the optimization of the robot’s movement. This optimization process encompasses not only path planning but also speed determination and safe navigation [17]. In general, research conducted in simulation environments represents a major driving force behind the successful development and practical applicability of physical pruning robots. Current research and development trends indicate that the integration of viticultural requirements, robotics, and information technology (IT) will form the foundation of the next generation of automated vineyards [13,18].

The primary objective of this study is to highlight the development value of a digital-twin-oriented virtual training environment for an autonomous agricultural robot capable of navigating between grapevine rows. Since virtual environments provide opportunities for training models that perform various tasks on robotic platforms, the training-oriented value of simulation is concrete. First, it provides a safe environment for evaluating key perception and navigation components, such as path planning, without exposing either the robot or the plants to unnecessary risk. A robot can fail, repeat, and improve in virtual space more safely, more cheaply, and more systematically than in a vineyard. This is especially important when real-world testing is constrained by seasonality, crop-damage risk, terrain variability, and limited access to expert operators. Second, it supports efficient data generation. In a virtual environment, large volumes of training data can be collected much faster than in real agricultural settings, where data acquisition is often slow, labor-intensive, and season-dependent. Third, simulation makes it possible to expose the robot to a wide range of environmental conditions in a controlled way. This improves model adaptability, reduces the risk of overfitting to a narrow set of field conditions, and can support more robust operation in later deployment stages. Although the adaptation of models that perform well in simulation environments to real-world conditions may still present challenges, the proposed digital-twin-oriented framework provides an appropriate basis for addressing these issues. The contribution of this paper is threefold:

- The concept presented in this paper reinterprets digital twin technology as an active robot-training environment rather than merely as a passive mirror of a deployed machine.
- This study proposes a layered framework for training agricultural robots in virtual environments, covering robot embodiments, sensing, terrain, tasks, and management-side feedback.
- A reinforcement learning-based agent is introduced for adaptive path planning within a simulated vineyard environment, where it successfully navigated to 155 target points out of 161.

The remainder of our work is structured as follows. Section 2 reviews the related literature on digital twins, robotics simulation, and agricultural robots. Section 3 presents the proposed methodology and the design principles of the digital-twin-based training framework. Section 4 describes the experimental protocol, including the evaluation scenario, baseline controller, neural network architecture, sensor channels, reward function, and curriculum-based training environment. Section 5 reports and discusses the experimental results. Finally, Section 6 concludes the paper and outlines directions for future work.

## 2. Related Work

General digital twin literature provides an important conceptual starting point. Kritzinger et al. [15] distinguish among the digital model, the digital shadow, and the digital twin, and show that the fully integrated twin is the most demanding stage of development. Jones et al. [16] further clarify the concept by identifying its characteristic components, including the physical entity, the virtual entity, the environment, the twinning connections, and the associated processes. These works established the digital twin concept and prevented the term digital twin from degenerating into a vague synonym for simulation.

Studies have already appeared in the literature in which the authors attempted to exploit the potential offered by digital twin technology. Liu et al. [19] simulated a dual-arm grape-harvesting robot and highlighted that development in real-world environments is hindered by several factors. These include seasonality and adverse weather conditions, which significantly limit the time window available for data collection. Another challenge is the reproducibility of experimental conditions within the same vineyard. Finally, during real-world operations, collisions may occur between the robot’s body or robotic arms and objects in the environment. The [20] paper applies a digital twin to a vineyard automation scenario to improve how stakeholders interact with data and decision-support tools. Instead of focusing only on technical modeling, the authors developed a human-centric framework that adapts the vineyard digital twin to different user roles (e.g., farm managers and operators) through tailored dashboards and cognitive modeling methods. The digital twin was used to support real-time monitoring, data interpretation, and operational decision-making, while reducing information overload and improving trust and usability. In [21], a digital twin–driven system has been proposed for indoor tomato harvesting. Images captured by a movable depth camera mounted on the robot are processed using the Unity engine to replicate the real environment in a virtual space. With the help of the digital twin, the tomato ripening process was simulated in real-time based on the collected data. Subsequently, a model was trained using data derived from the virtual environment to support the robot’s decision-making process.

In robotics, the distinction between simulation and digital twin should not be understood too rigidly. The useful question is not whether a model is already a complete digital twin in the strongest industrial sense. The useful question is whether it is rich enough to support meaningful experimentation, validation, and later deployment. A realistic simulator makes it possible to test kinematics, dynamics, sensing, and control in a controlled environment before taking risks in the field. Models trained in simulation and subsequently fine-tuned using real-world data demonstrated performance comparable to models trained entirely on real datasets [13]. This represents a significant advantage in the development of pruning robots, where real-world testing can be both costly and potentially risky in terms of yield loss. In sim-to-real literature, this idea is strengthened by methods that deliberately widen the variability of training conditions. Domain randomization and related techniques seek policies that remain useful across a distribution of conditions rather than a single idealized simulator state [22]. This is directly relevant for outdoor robots, whose real working conditions are never as clean and stable as a laboratory benchmark.

The motivation for agricultural robots is now well established. Marinoudi et al. [6] argue that robotics and automation can help mitigate both year-round and seasonal labor shortages in agriculture. Yang et al. [7] describe robotization as one of the effective responses to the increasing shortage of skilled labor in crop production and review the core technologies needed for mobile platforms, actuation, perception, and operation planning. Liu et al. [8] reach a similar conclusion at the field-robot level and emphasize that future agricultural robots will require intelligent sensing, smart decision-making, and advanced integration technologies, including digital twins.

A recent technical review by Spagnuolo et al. [14] reinforces this interpretation by presenting agricultural robotics as a system-level response to the challenges of sustainable crop production. This is close to the position adopted in the present paper. The agricultural robot is not merely a mobile platform with a single end-effector. It is part of a broader technical system in which sensing, movement, task execution, environmental adaptation, and decision support must work together under real field constraints.

For vineyards, an important question is how pruning operations performed manually can be replaced by mechanized and eventually automated solutions. For a long time, this goal appeared unattainable because the necessary technological background was missing. Navone et al. [13] show that significant research attention to autonomous robotic pruning in orchards and vineyards emerged only in the late 2010s. This historical observation is significant because it places current pruning research in the context of a rapidly maturing field rather than a long-established engineering routine.

The development of a pruning robot requires the solution of several general and task-specific subtasks, such as cutting-point estimation [23,24], tree skeletonization [9], and path (route) planning [18]. Out of those subtasks, the present study primarily focuses on route planning in the virtual environment with advanced obstacle avoidance. For indoor navigation, in most cases, a two-dimensional (2D) camera alone is sufficient for following a predefined path [25,26]. However, under uneven terrain conditions, sensors capable of generating depth information, such as stereo cameras and LiDARs (Light Detection and Ranging), provide a more effective solution for accurate route planning of robotic systems [27]. As an example, the authors of [28] present the design concept of a robotic system developed for grape harvesting and defoliation. Navigation within the plantation is based on a map defined by GPS coordinates. Their row-following navigation primarily relies on measurement points obtained from the LiDAR sensor, complemented by signals from wheel encoders and inertial sensors. The authors note that obstacle avoidance within the narrow grapevine rows represents a significant challenge for the robot. Based on this limitation, they assume ideal operating conditions in which no obstacles are present between the grapevine rows that would require avoidance.

Although simulation environments offer valuable opportunities for data collection and model training, in practice, such models will perform effectively only if the simulated environment accurately represents real-world conditions. Botta et al. [29] pointed out that power balance, traction allocation, and terrain interaction matter for sustainable agricultural monitoring robots. This observation has a direct implication for training. A digital twin that teaches navigation or mission planning while abstracting physically important locomotion effects will teach the wrong lesson.

Even though a few studies have already attempted to exploit the potential of digital twin technology for the development of agricultural robots [17,20,21], digital-twin-supported robot development in viticulture remains a relatively new area. The available works show that integration is beginning to appear around autonomous navigation, monitoring, and task-oriented operation, but the training-oriented interpretation of the digital twin is still less explicit than it could be. This is one of the reasons why viticulture is a useful case for the present paper. It is technologically advanced enough to support digital twin development, yet still open enough that the systems-level role of virtual training deserves clearer formulation.

## 3. System Architecture and Virtual Training Framework

We define a reproducible digital-twin-based workflow in which robot control, sensor simulation, virtual scenario generation, and model transfer to physical deployment can be organized coherently. The central methodological assumption is that the digital twin should function as an active virtual training environment rather than as a static visualization artifact. This interpretation follows the broader digital twin literature, which treats the twin as a structured virtual counterpart of a physical system rather than a mere geometric model [15,16].

Accordingly, the present methodology is built around five design principles. First, the control data must run through the Robot Operating System (ROS) side as much as possible. Second, the Unity environment should provide the digital twin of the robot, the terrain, the plants, and the relevant task context. Third, communication between ROS and Unity should be interface-driven, so that the ROS-side software can be exercised first in the digital twin and later against the physical robot. Fourth, the simulation environment should be rich enough to support safe testing, efficient data generation, and variation in training conditions. Fifth, the transfer from simulation to the physical platform should be staged and explicitly calibrated, rather than assumed to be automatic.

### 3.1. Overall System Architecture

The proposed system architecture contains five main blocks (Table 1): (i) a physical robot, (ii) a ROS-based software layer, (iii) a Unity-based digital twin, (iv) a bidirectional communication ecosystem between ROS and Unity, and (v) an evaluation layer.

**Figure 1:**
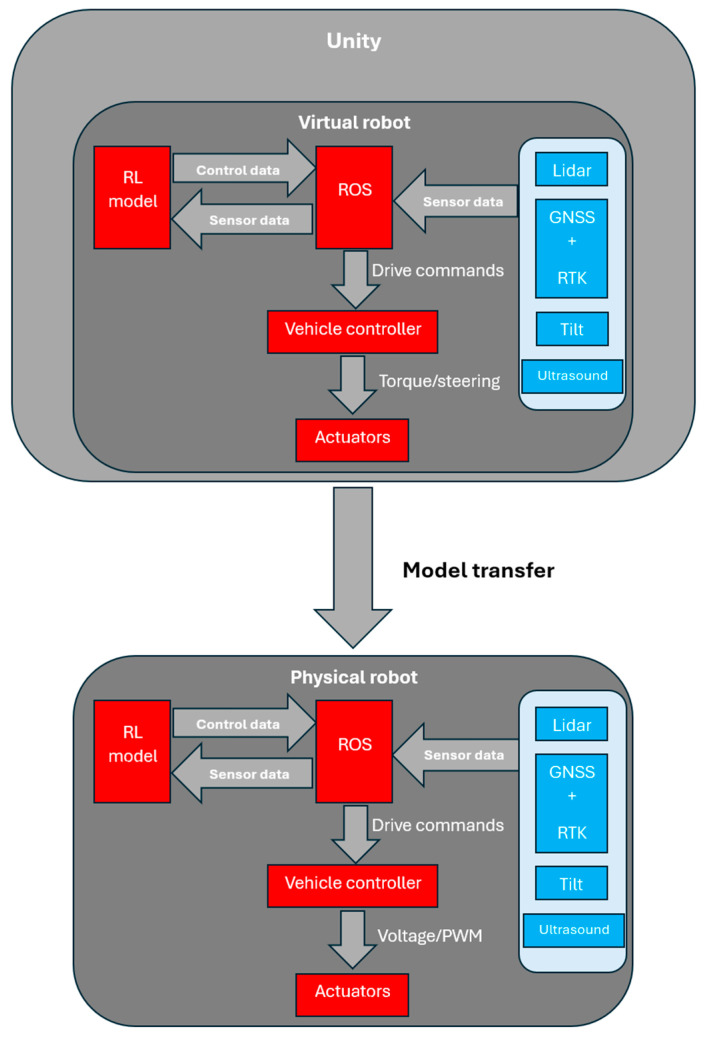
A simplified block diagram of the proposed system architecture where the reinforcement learning (RL) model is responsible for the autonomous navigation of the pruning robot (See Section 4.4). The sensor layer incorporates the LiDAR and the Global Navigation Satellite System receiver with Real-Time Kinematic support (see Section 3.3.5). The evaluation layer is not shown.

The physical robot contains real actuators, sensors, and onboard or edge-connected computing resources. The ROS layer contains control logic and communication interfaces for sensors. The Unity layer contains the virtual robot, the simulated sensors, the terrain and crop environment, and the task-oriented scene logic. The communication ecosystem maps ROS messages to Unity-side interfaces and returns simulated observations to the ROS side [30]. The evaluation layer is responsible for logging and metric computation. For illustration purposes, a simplified block diagram of the proposed system architecture can be seen in Figure 1.

### 3.2. ROS-Based Control Layer

ROS 2 was selected as the main software backbone because it provides a mature distributed framework for robot applications, including nodes, topics, services, actions, and interface-based interoperability. In the present methodology, the ROS layer is expected to contain at least the following functional modules:

- A motion-control module for low-level command generation.
- A perception module receiving LiDAR, and other sensor streams.
- A navigation module for route following and local reaction.
- A logging and evaluation module for recording virtual runs.

The methodological advantage of this arrangement is the separation of concerns. The robot logic is expressed in ROS modules, while the execution may be virtual or physical. This keeps the training workflow transparent and supports later debugging, because failures can be localized either to the logic layer or the embodiment layer.

### 3.3. Unity-Based Digital Twin Environment

Unity was selected as the main simulation platform because it combines general-purpose 3D scene construction, physics-based interaction, extensibility, and official robotics-oriented tooling [30,31]. The same ecosystem also provides ROS connectivity and Unified Robot Description format (URDF) import support, which are particularly valuable when the virtual robot must remain structurally aligned with the physical platform [32,33].

In the proposed methodology, the Unity environment contains both the virtual robot and virtual agricultural scenes. The robot side includes geometry, kinematic structure, motion constraints, and simulated sensors. The environment includes terrain, grapevine row or lane structure, plants, obstacles, refill or service stations where relevant, and configurable lighting or visibility conditions. The goal is not to reproduce every physical property at maximum fidelity, but to represent the properties that are relevant for the training tasks under study.

A vineyard-oriented rover scenario is used as a representative case, in line with previous vineyard robot development work in which navigation, sensing, route planning, and farmer-side support are treated as parts of one integrated robotic workflow [34].

#### 3.3.1. Realistic Locomotion Modeling in the Unity-Based Digital Twin

In vineyard and field robotics, the robot does not move on ideal laboratory surfaces, but on uneven terrain shaped by slope, roughness, loose soil, ruts, and local discontinuities. For this reason, the mobility layer of the digital twin must represent those wheel–terrain interactions that materially influence navigation, obstacle avoidance, and task execution. If these effects are abstracted too aggressively, the virtual environment may still look plausible, but it will teach the wrong control and validation lessons.

This issue is particularly relevant in Unity-based simulation, where the built-in “WheelCollider” component offers a convenient baseline solution but relies on a simplified contact model. The “WheelCollider” simulates wheel–ground interaction by casting a single ray downward from the wheel center and deriving suspension and slip forces from that point-based contact estimate. This makes it computationally efficient and convenient for many vehicle prototypes, but it cannot represent multi-point or surface-like wheel contact.

In practice, this means that wheel–terrain interaction is reduced to a single-ray approximation, which may be sufficient for some game-style use cases, but becomes problematic when a robot must traverse sharp edges, stairs, grates, or loose obstacles. Under such conditions, the simulator may produce implausible behaviors, including sudden drops into narrow gaps, unstable climbing on vertical steps, or unrealistic impulses during contact with lightweight objects. These artifacts are especially undesirable in digital twin scenarios, where the purpose of simulation is not entertainment but the development and validation of robotic behavior.

For this reason, a more suitable approach for agricultural robotics is to model locomotion with a stronger emphasis on physically meaningful wheel contact. A possible solution may be based on the “ConfigurableJoint” component, since it allows the wheel body, its collider, and its connection to the chassis to be modeled more explicitly than in a single ray “WheelCollider” setup. In such a configuration, each wheel can be represented as a “Rigidbody” connected to the vehicle body through a “ConfigurableJoint”, with colliders on both the wheel and the chassis so that contact is resolved through the actual wheel geometry rather than through a single probing ray. This also makes it possible to tune angular drives, motion constraints, and mass scaling in a controlled way, so that the wheel remains stable, follows its intended rotation smoothly, and interacts more robustly with irregular terrain.

#### 3.3.2. Skid-Steered Rover Motion

In the present work, the steering model of practical interest is skid steering. Skid steering is a locomotion method in which the left and right sides of the vehicle are driven at different speeds, or in opposite directions, in order to generate curved motion or on-the-spot rotation. Its main advantage in robotic applications is maneuverability in constrained spaces, since the vehicle can perform tight turns without relying on a dedicated steering linkage.

Turning performance depends directly on traction and on the interaction between the wheels and the ground surface. On a smooth idealized plane, differential drive commands may appear easy to reproduce, but on sloped or irregular terrain, the same commands can yield substantially different motion. For this reason, a virtual rover should not merely receive abstract “turn left” or “turn right” commands. Instead, the Unity-side embodiment should express skid-steered motion through differential actuation of the vehicle’s sides, so that curved driving, in-place rotation, and traction-dependent turning emerge from the locomotion model in a more realistic way.

A practical implementation route is to use a wheel representation in which the wheels are connected to the chassis through configurable physical constraints, while the controller maps forward and turning intent to different left and right-side wheel velocities or torques. In such a setup, the digital twin can express straight motion, stationary turning, and mixed turning-while-moving behavior within one consistent control model. This is methodologically useful because the same high-level ROS-side logic can be exercised first in simulation and later against the physical rover.

#### 3.3.3. Procedural Terrain Generation for Vineyard Training Scenarios

Terrain generation should support controlled variability in slope, roughness, traversability, and scene layout. In an agricultural digital twin, this is methodologically valuable because it reduces the risk that the robot is optimized for a single clean virtual field instead of learning to operate across a broader distribution of conditions. The framework already identifies terrain geometry as one of the core dimensions of scenario variation, alongside obstacles, plant distribution, visibility conditions, and mission layout.

For the terrain surface itself, Perlin-noise-based height generation provides a practical solution [35]. A single noise layer is usually too smooth for a convincing field-like environment; therefore, multiple octaves can be combined to create larger and smaller terrain features simultaneously. In this setup, lacunarity controls the increase in frequency across octaves, while persistence controls how strongly the higher-frequency detail contributes to the final surface. The resulting heightmap can then be converted into a procedural mesh by generating vertices on a grid, connecting them through triangles, and assigning the mesh to the terrain object. When a “MeshCollider” component is added, the generated surface becomes physically traversable for the simulated rover. A “MeshCollider” allows the physics engine to treat the generated mesh itself as a collision surface, so wheel and body contacts are resolved against the actual terrain geometry rather than against a simplified placeholder. This is particularly important in robotics-oriented simulation, because reliable terrain contact is needed not only for visual plausibility but also for stable and predictable vehicle motion during training and validation.

#### 3.3.4. Obstacle Placement and Grapevine Row Generation on Uneven Terrain

After the terrain surface has been generated, the next methodological step is to populate it with task-relevant structure. In a vineyard-oriented digital twin, this means more than adding arbitrary visual objects. The environment should contain grapevine row organization, plant placement, navigable inter-row space, and obstacle patterns that reflect the operational conditions of the rover. Since the framework already treats obstacle placement, plant distribution, and mission layout as separate dimensions of scenario generation, it is natural to formalize this stage as a dedicated environment-construction step.

A useful design principle is to combine deterministic and stochastic placement. Grapevine rows should be generated in a structured manner because their spacing, direction, and continuity define the navigational logic of the task. On uneven terrain, this can be implemented by first defining row centerlines or row anchor points in the horizontal plane, then projecting the generated row elements downward onto the actual terrain surface. In practice, this allows row geometry to remain globally coherent while still conforming to local height variation. Such an approach is especially suitable when the environment must preserve recognizable vineyard structure but still reflects the physical irregularity of hilly ground.

Obstacle placement can then be added on top of this structured layout in a controlled stochastic way. Poisson disk sampling is particularly useful here because it avoids unrealistic clumping while still producing a non-uniform and natural-looking distribution [36]. This is valuable not only for visual plausibility but also for training quality: a robot should encounter obstacles, vegetation, or waypoints in a field that is challenging and varied but not distorted by accidental overlaps or extreme density spikes caused by naive random placement. The same logic can be used for auxiliary task objects, such as markers, service points, or secondary vegetation elements, provided that exclusion zones are maintained around essential navigation corridors or row segments.

#### 3.3.5. Sensor Simulation in the Unity-Based Digital Twin

For agricultural rover development, Unity must provide sensor outputs that are sufficiently informative for the ROS side perception and control modules, while remaining configurable enough to reflect changing field conditions. The framework already takes this position explicitly by treating the sensor layer as an operational component of the training workflow, with Unity-generated observations fed back to the ROS side through the integration bridge.

LiDAR simulation is equally important in a vineyard scenario, especially when the rover must detect obstacles, estimate free space, and maintain reliable local navigation under changing terrain and visibility conditions. In Unity, LiDAR-like sensing can be approximated through raycasting from the sensor’s origin into the virtual scene. By controlling angular spread, range, angular resolution, and refresh rate, the simulator can emulate different sensing profiles and generate point-cloud-like observations from the resulting intersections. Additional realism can be introduced through noise, occlusion, and material-dependent variation, since real LiDAR sensing is affected by blocked rays, imperfect surfaces, and environmental disturbances.

Beyond LiDAR channels, the digital twin may also expose GNSS (Global Navigation Satellite System) based on GPS (Global Positioning System) with Real-Time Kinematic (RTK) localization and task-specific internal state variables, such as tool state, or mission flags. The GPS/GNSS position is represented in the virtual environment by the rover’s displacement in the Unity world, which is continuously tracked.

#### 3.3.6. Design Trade-Offs and Task-Relevant Fidelity

A Unity-based agricultural digital twin should not be judged primarily by whether it reproduces every physical detail of the real robot and field at maximum fidelity. Such a goal would often be impractical and, in many cases, unnecessary. A more useful design principle is task-relevant fidelity: the simulation should model those aspects of the system that materially influence the training, validation, and transfer of the target robotic behavior. This principle is already implicit in the framework, which states that the virtual environment should represent the properties relevant to the training task rather than all physical properties indiscriminately.

In the present context, this means that some components deserve higher modeling priority than others. For example, wheel–terrain interaction is critical if the rover must navigate hilly vineyard-like ground, because unrealistic locomotion may distort both control learning and performance evaluation. Row geometry, obstacle spacing, and mission layout also have direct methodological importance because they define the structure of the task presented to the robot. By contrast, many low-level electromechanical details of the real platform may be omitted or simplified if they do not materially affect the observations and state transitions relevant to the training objective.

### 3.4. ROS–Unity Integration Mechanism

Unity runs as a standalone application connected to ROS through explicit transport tooling. On the Unity side, message exchange is handled through the “ROS-TCP-Connector” package [32,37]. The Unity Robotics Hub documentation further shows that this integration model is intended to support message exchange, topic publication/subscription, and staged transfer from simulation to real-robot execution [38].

In the present methodology, the connection between the components has three roles. First, it transmits simulated observations, such as sensor messages or state information, from Unity to ROS. Second, it returns control commands from ROS to the simulated robot. Third, it preserves interface compatibility between virtual and physical operations. In practice, this means that the ROS-side software can be tested against simulated LiDAR, odometry, or task-state channels before the same logic is connected to the corresponding physical interfaces. Furthermore, the same high-level control intent can be transmitted through ROS in both simulated and physical execution. In the Unity-based digital twin, such commands are interpreted as simulated actuation inputs according to the game engine physics, whereas on the physical platform, they are mapped to the corresponding motor-level commands.

We therefore make the following cautious claim: when topic, service, and message contracts are preserved, the same ROS-side nodes and launch structure can be exercised first against the Unity-based digital twin and later against the physical robot.

The URDF pathway is also important here because the official URDF Importer from Unity Technologies enables URDF robot models to be brought into Unity as native simulation objects [33]. Methodologically, this reduces duplication and supports consistency between the ROS-side robot description and the Unity-side digital twin.

### 3.5. Unity–Python Control and Training Interface

In addition to the ROS–Unity integration described above, the virtual training workflow also relies on a Unity–Python control interface for neural policy execution and training. In this setup, Unity serves as a simulation environment, which can run either in real time or at an accelerated simulation rate. For training, this can be controlled through Unity’s time-scaling mechanism and, in ML (Machine Learning) Agents workflows, through the “time-scale” option. This architecture enables closed-loop interaction between the virtual robot and the learning-based controller.

On the Unity side, the correct robot configuration includes the control script and the standard ML-Agent-related components, such as Agent, Behavior Parameters, and Decision Requester. On the Python (3.13) side, the external runner must match the observation and action specifications expected by the Unity environment.

The execution order of the pipeline is also methodologically important. The Unity project must first compile correctly, load the ML-Agents-enabled robot hierarchy, and enter “Play” mode without excessive logging or interface mismatch. Only after this can the external Python process attach successfully and start policy inference or training.

The practical significance of this interface is that it defines a reproducible bridge between the digital twin and the control layer. It also makes it possible to distinguish clearly between simulation-side problems, interface mismatches, and policy-side problems during debugging and validation.

### 3.6. Virtual Training Workflow

The virtual training workflow is organized as a staged loop rather than a one-shot simulation. The steps are the following:

- Define the robot task and the corresponding ROS-side interfaces.
- Construct or import the robot model into Unity.
- Configure the connection between ROS and Unity.
- Create the virtual agricultural scene and its scenario parameters.
- Run repeated virtual trials to generate data, test control logic, or train learning-based modules.
- Record logs, failures, and performance metrics.
- Refine the virtual model, ROS parameters, and repeat.

Virtual operation provides a safe setting for evaluating key functions, avoids unnecessary risk to plants and hardware, and enables efficient data generation under diverse conditions. It also supports trust building: even a skeptical farmer is more likely to accept a robot in real field conditions if it has already demonstrated reliable behavior in virtual fields. This is one of the principal reasons why the digital twin is treated here as a training space rather than as a mere visualization artifact.

The first transfer principle is interface preservation: the ROS-side logic should communicate through channels that exist in both virtual and physical systems. The second principle is staged deployment: modules are moved to physical execution incrementally, beginning with logging and replay, then supervised control, and only later full closed-loop trials. The third principle is explicit calibration: differences in timing, noise, localization accuracy, wheel-terrain interaction, or actuation delay are identified and corrected where practical.

### 3.7. Virtual–Physical Correspondence and Accuracy Assurance

The simulated rover was designed to correspond to the physical platform in the properties that directly affect navigation and control. It reproduces the real robot at a one-to-one scale in terms of geometry, shape, mass, wheel configuration, and sensor placement. Wheel–terrain interaction is handled by a custom full-mesh-based component, allowing the wheels to interact with the actual terrain mesh rather than relying on a simplified single-ray wheel model.

No explicit actuation delay is currently modeled. Quantitative field-level validation remains future work and will compare simulated and real trajectories, sensor data, target-reaching accuracy, and command-to-motion delays in order to assess the accuracy of the digital twin under real operating conditions.

## 4. Experimental Protocol

This section describes the experimental protocol used to evaluate the proposed virtual training framework. The quantitative results and their interpretation are reported separately in Section 5.

### 4.1. Experimental Platform and Evaluation Scenario

**Figure 2:**
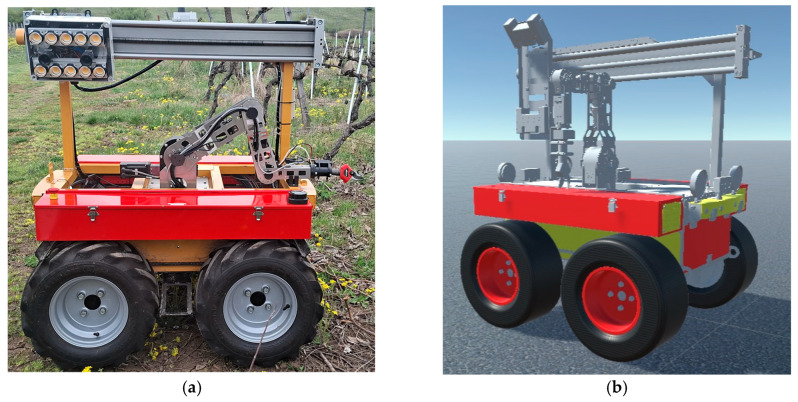
(**a**) The physical robot; (**b**) the digital twin.

As a reference, Figure 2a shows our “Grapler” grapevine-pruning robot prototype [34] while Figure 2b shows its digitalized copy. The correspondence between the physical and virtual representations is close, but not perfectly identical, because the two figures reflect slightly different stages of development. In particular, the digital twin still contains an earlier camera configuration, whereas the physical robot has no illumination unit. The platform combines a mobile rover base, a robotic arm equipped with a pruning shear, a rail-mounted camera and a high-intensity illumination unit, two 2D cameras mounted at an angle for environment digitization, protected onboard electronics, an emergency stop button, LiDAR, and GPS/GNSS-based localization with Real-Time Kinematic (RTK) correction. RTK correction technology enables centimeter-level positioning accuracy, typically in the range of a few cm under favorable conditions.

**Figure 3:**
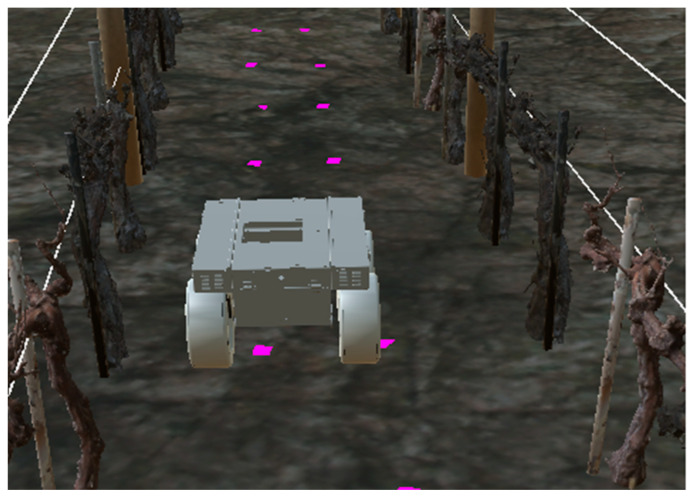
The virtual “Grapler” rover: self-driving rover in the Unity-based vineyard.

Figure 3 shows the virtual self-driving rover (without the camera system and robotic arm) in the Unity-based agricultural environment. The scene represents a vineyard-like training field with grapevine row structure, plant objects, target markers, and a mobile rover placed between the grapevine rows. The virtual rover is used to train and validate autonomous navigation before the corresponding logic is considered for physical deployment.

### 4.2. Self-Driving Task in the Digital Twin

In the virtual task, the rover was required to navigate autonomously to task-relevant locations while avoiding collisions and maintaining stable progress in the environment. The task took the form of a vineyard pruning-related navigation scenario: the rover had to reach plant positions specified by GPS/GNSS coordinates while avoiding obstacles and contact with the plants. The robot must move toward a goal, interpret sensor information, and avoid obstacles. In this sense, the pruning scenario is more informative than a pure point-to-point navigation task. It tests whether the digital twin can support a complete autonomous behavior loop. This confirms that the scenario is not only a conceptual model, but an executable virtual training setup in which a rover agent can be controlled from the learning side and evaluated in the Unity environment.

### 4.3. Baseline Controller

To establish a performance benchmark, we implemented a reactive heading-based controller. The controller operates as a closed-loop system. The controller continuously computes the angular difference between the rover’s current yaw (ψ_rover_) and the bearing to the target waypoint (ψ_target_). To account for the angular discontinuity at ±π, the heading error is normalized using a shortest-path formulation, ensuring that the resulting error remains within the range [−π,π]. This guarantees that the controller always selects the minimum angular rotation required to align the rover with the target direction. The steering command becomes the angular difference clipped to [−1, 1] range.

### 4.4. Artificial Neural Network Architecture

Although more techniques exist for implementing intelligent robot control [39], the objective was to perform this task using a data-driven model. Therefore, the autonomous behavior of the rover was produced by a neural network trained with the help of the Unity ML-Agents pipeline. In the project configuration, the policy operated on a 24-dimensional observation vector while producing a 2-dimensional continuous action vector. The observation space included navigation-relevant and perception-relevant quantities, such as heading error, velocity, target distance, and LiDAR-derived features from the virtual environment. The elements of the final vector can be found in Table 2. The action space encoded longitudinal motion and turning commands.

The self-driving behavior was not created through manually scripted waypoint logic, but through a learned policy that mapped structured observations to motor-relevant control actions in a closed loop. The Unity environment provided simulation and sensor streams, while the policy execution and training pipeline were coupled through the ML-Agents runtime and the external Python side.

The neural network is a Proximal Policy Optimization (PPO) [40] implementation. The exact architecture, including hidden-layer structures, activation functions, and optimizer settings, can be read in Table 3.

### 4.5. Sensor Channels Supporting Autonomous Navigation

Autonomous driving in the digital twin relied on multiple sensor channels. The main channels were LiDAR-based obstacle sensing, GPS/GNSS-based localization, and motion-state information. Among these, the GPS/GNSS channel had a particularly important role because the rover had to navigate toward task-relevant waypoints.

The LiDAR and ultrasonic sensor channel provided geometric information about obstacles and free space, supporting safe local navigation, especially in cluttered or narrow areas. The GPS/GNSS channel provided global or semi-global localization information and therefore played a central role in waypoint following and return-to-station behavior. Motion-state variables, such as velocity and heading-related information, helped stabilize control. LiDAR contributed most directly to local safety because it exposed nearby obstacles and traversable space. The GPS/GNSS channel contributed most directly to reliable global positioning and mission-level navigation, especially when the rover had to move between spatially separated targets or return to the refill point. The digital twin made it possible to test these channels together without risking hardware or crops.

Most of the sensors were simulated using Unity’s built-in state variables. The GPS data were derived from the coordinate system and position variables. Speed can be obtained from the rover’s Rigidbody velocity. The tilt angles are the rover’s transform “EulerAngles” along the respective axes. The virtual LiDAR sensor was configured to mimic the specifications of industrial-grade 2D scanning units common in agricultural robotics. The sensor is simulated using “Raycasts” and provides a 360-degree field of view, covering an angular range from 0 to 360 degrees with an angular resolution of 0.12 degrees, yielding 3000 discrete range measurements per full scan. The detection range is constrained to an operational interval of 0.1 m to 5.0 m, reflecting the typical proximity requirements for safe inter-row navigation and obstacle avoidance in vineyard environments. Ultrasonic sensors are forward-directed “SphereCasts” with distance-based dynamic radii scaling. Two sphere casts with different radii were used to emulate beam spread and detection sensitivity, and the minimum detected distance was returned as the sensor measurement.

### 4.6. Reward Function

The reward function was designed to encourage the rover to navigate toward a specified GPS target while avoiding collisions and unstable vehicle states. A large negative terminal reward is assigned when the episode ends in failure, which occurs if the rover collides with an obstacle, tips over, or exceeds the maximum episode duration of 2000 simulation steps (equivalent to 50 s of simulated time). Conversely, a large positive terminal reward is assigned when the rover successfully reaches the target location, defined as being within 0.1 m of the goal.

In addition to the terminal rewards, the agent receives dense intermediate rewards at each timestep. Progress toward the target is encouraged by rewarding reductions in the distance to the goal and penalizing increases in distance relative to the previous timestep. To promote efficient navigation behavior, turning is penalized when the rover is already well aligned with the target direction, while forward motion is penalized when the rover is not oriented toward the goal. These shaping rewards encourage the agent to first align itself with the target before advancing and to maintain consistent progress toward the destination.

### 4.7. Curriculum-Based Training Environment

The training process was structured progressively as a curriculum consisting of four environments of increasing difficulty. This ensures that the agent gradually learns harder tasks, building prior knowledge, instead of immediately tackling the most difficult task, which may be impossible from a random initialization. The idea was to take the rover on the harder map each time it successfully reaches the target location in at least 90 percent of the last 200 episodes.

The first environment is a flat plane with no obstacles, where the agent learns how to navigate to given points. To avoid overfitting specific trajectories, the starting location, the starting rotation of the rover, and the given endpoint are randomized. The second environment introduces obstacles in the form of walls. This way, the agent can learn to avoid them while going to the target point. The walls are placed on each side of the rover, spanning from the rover to the target, imitating a grapevine row. The third environment changes the flat plane to an uneven terrain with elevation changes. The target points are still randomized at the beginning of each episode, but the starting point of the rover is the last reached target point. This way, the agent can operate continuously without interruptions. The fourth environment is aimed at being more realistic than the previous environments. The walls are replaced by grapevine rows. There is also a demo environment that is used for evaluation only and is designed to assess the generalization ability of the agent with a more realistic task. It consists of three different grapevine rows of varying lengths. One on a flat part of the terrain, one going uphill, and the last one running along the slope. To increase terrain complexity, there are smaller rocks placed inside the grapevine rows using Poisson disk sampling. Each episode lasts until the rover reaches a terminal state or runs out of the allocated maximum time of 2000 timesteps. Terminal states are reaching the target position, colliding with something, or flipping over. Unity’s base simulation speed is 50 timesteps per second, but it can be increased using the project’s “TimeScale” parameter.

The third environment uses a single Perlin noise map scaled down with a factor of 0.25 to introduce smaller hills. For the fourth training and for the demo environment, a procedurally generated map was used. The generation is also based on Perlin noise. To achieve realistic terrain topology, we employed a multi-octave Perlin noise approach. The terrain heightmap is generated by the superposition of multiple noise octaves, where higher-frequency layers provide small-scale surface roughness, while the base layer defines the large-scale hill structure, allowing for systematic variation in surface topography. We define the landscape geometry using 8 octaves with a persistence of 0.454 and lacunarity of 2. The vertical scale is governed by a height multiplier of 10, resulting in maximum elevation variances of approximately 10 m. This procedural pipeline ensures that the rover encounters varying gradients and roughness levels during training, fulfilling the requirement for a varied, non-trivial training distribution.

**Figure 4:**
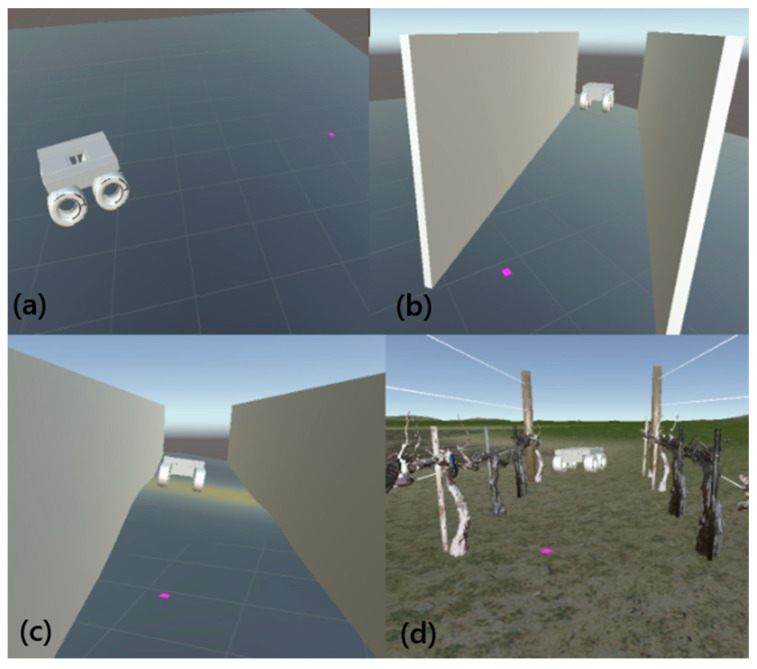
The four training environments: (**a**) a flat terrain without obstacles, (**b**) a flat terrain with wall obstacles, (**c**) an uneven terrain with elevation changes, and (**d**) a more realistic vineyard-like environment.

**Figure 5:**
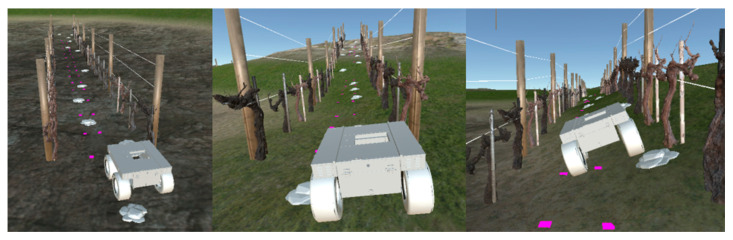
The three grapevine rows in the demo environment.

For demonstration purposes, Figure 4 shows the four training environments used in the curriculum-based learning process: (a) a flat terrain without obstacles for basic target reaching, (b) a flat terrain with wall obstacles for obstacle avoidance, (c) an uneven terrain with elevation changes, and (d) a more realistic vineyard-like environment in which the walls are replaced by grapevine rows. Figure 5 shows the demo environment used for evaluation, consisting of three grapevine rows with different terrain characteristics: one on flat ground, one on uphill terrain, and one along a side slope.

Catastrophic forgetting [41] is the phenomenon where a neural network’s performance worsens earlier tasks when learning a new task. Taking this into account, the second and third training environments alternate every 100 episodes between configurations with and without walls. This approach allows the agent to learn about wall avoidance while retaining its ability to navigate without walls.

Each stage of the training process uses only the sensors that are required for task completion. Subsequent stages build upon the pretrained model by adding additional sensor inputs. The first stage uses the angle between the rover’s forward direction and the direction to the target point, as well as the rover’s velocity. The second stage uses LiDAR sensor measurements. The third stage adds tilt angles and ultrasonic sensor measurements.

The training process is incremental. The model is random at the beginning of the first environment. After it converges into a stable policy, additional sensor modalities are introduced into the observation space, and training is continued using the weights of the previously trained model as initialization. This is repeated for each training environment.

## 5. Results and Discussion

The results of the present study are centered on one practical question: Can the digital twin function as a useful virtual training environment for self-driving agricultural robots? In order to answer this question, we focus on autonomous navigation, the sensor channels that support it, the staged training process carried out in the virtual environment, and the role of realistic locomotion in validating the learned behavior.

The main result at this stage is that the digital twin provided a sufficiently rich execution space for autonomous mission behavior. The rover was not restricted to scripted playback. Instead, the virtual environment supported online decision-making based on simulated observations, which allowed the self-driving pipeline to be trained and repeatedly exercised under controlled but non-trivial conditions. This supports the interpretation that the proposed digital-twin-oriented virtual environment can function as an operational training environment for autonomous navigation rather than merely as a visual demonstration.

### 5.1. Training Progress

**Figure 6:**
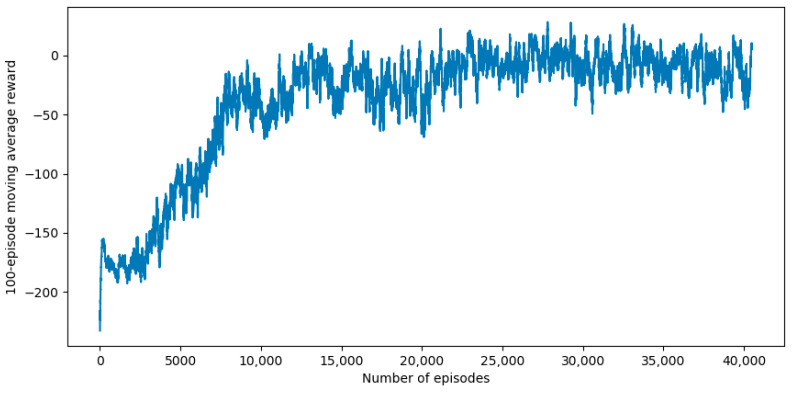
Training curve for the first training environment.

The duration of training varied across environments depending on the complexity of the task and the convergence rate of the policy. Convergence was achieved after approximately 40,000, 44,000, 30,000, and 45,000 training episodes for the first, second, third, and fourth environments, respectively. As an example, Figure 6 shows the training curve of the agent for the first training environment, where the horizontal axis shows the number of episodes, while the vertical axis shows the average reward in the last 100 episodes.

### 5.2. Comparative Evaluation Against the Baseline

Five independent evaluation runs were conducted for both the baseline and the trained RL controller under identical simulation conditions. Table 4 summarizes the success rate and failure types across all five runs. When the robot became stuck, it could not reach the next target point within one minute.

The RL controller achieved higher success rates and fewer collisions than the baseline in all three grapevine row types. However, the baseline controller completed successful movements faster in some cases, which indicates a trade-off between navigation safety and travel time.

Table 5 shows the summary of distances between target points in meters and the time it took for the agent to travel that distance in seconds. These values are based on successful episodes only.

### 5.3. Generalization in Procedurally Generated Scenes

A further result concerns generalization. The rover was not trained only in one manually fixed environment. Instead, procedurally generated layouts were used so that the robot repeatedly encountered new spatial configurations. This variation affected plant placement, obstacle placement, target distribution, and mission geometry.

The virtual scene uses structured randomness in the placement of objects. This is important because a self-driving agricultural robot should not memorize one path. It should learn behavior that remains useful under many similar but non-identical field layouts. Procedural generation made it possible to expose the rover to such variation at low cost.

The main outcome here is that the digital twin supported generalization-oriented training rather than route memorization. The policy had to cope with changing local conditions while preserving the overall mission objective. This is especially important in agriculture, where real field conditions are inherently variable. A policy that succeeds only in one idealized training scene is of limited practical value.

The importance of this result is consistent with the broader sim-to-real logic described in the literature, where robustness across a distribution of scenarios is preferable to optimization for a single clean environment [22]. In the present study, procedural scene generation widened the training distribution and made the digital twin a more meaningful preparation space for later real-world deployment.

### 5.4. Role of Locomotion Realism in Self-Driving Validation

The self-driving results also showed that autonomy cannot be evaluated independently of locomotion realism. This is particularly visible on uneven terrain. A navigation policy may appear effective if the underlying wheel–terrain interaction is oversimplified, even though the same policy becomes unstable once physically more realistic motion effects are introduced.

In the present workflow, uneven-terrain validation highlighted the importance of realistic contact behavior. The self-driving stack remained meaningful only when the digital twin produced believable mobility constraints. If the locomotion layer slips, bounces, or traverses terrain implausibly, then the navigation result is not trustworthy. For this reason, self-driving results should not be interpreted as perception-only or planning-only outcomes. They are system-level outcomes that also depend on the embodiment model.

This observation is consistent with the agricultural robotics literature, where terrain interaction, traction, and mobility are not secondary details but essential parts of field performance [29]. In the context of the present study, the practical lesson is clear: a digital twin that is intended to train autonomous driving must represent not only targets and obstacles, but also physically relevant mobility effects.

### 5.5. Transfer-Oriented Validation

The result is transfer-oriented rather than transfer-complete. This study does not claim that the simulation-to-reality problem has been fully solved. However, it does show that the digital twin supports a meaningful preparation step for physical deployment. The same ROS-side control logic could be exercised first against the virtual environment and then interpreted in a transfer-oriented manner for later physical validation.

## 6. Conclusions

This paper argued that digital twin technology should be understood in agricultural robotics not only as a representational model, but also as a virtual training environment. On this basis, it presented a methodology for designing a digital twin framework for teaching robots, including system architecture, ROS–Unity integration, virtual environment design, training workflow, and transfer-oriented evaluation. The vineyard rover case study then illustrated how this framework can support the development of self-driving behavior in a controlled setting. Meaningful behavior emerged from the interaction of several design choices: the use of multiple sensor channels, the curriculum-based increase in task difficulty, and the procedural variation in the environment. Together, these elements reduced the risk of training a policy that works only in one clean benchmark scene. The resulting interpretation is consistent with the broader argument of this paper: a useful digital twin must expose the robot to structured variation rather than to a single idealized world.

A further lesson concerns system design. The methodology kept ROS visible as an important design direction for modularity, interface preservation, and later transfer to the physical platform, while Unity provided the digital embodiment and the training environment. At the same time, the concrete learning workflow also depended on the Unity–Python connection and the ML-Agents pipeline. The practical value lies in the combination of a reusable system architecture, an operational digital twin, and a staged training workflow.

The results support the central claim of this paper: in agricultural robotics, a digital twin is most valuable when it is treated as an active training environment rather than as a passive mirror of a physical machine. In the presented vineyard case study, the virtual environment was not used only for visualization, but for iterative development of autonomous navigation, sensor interpretation, and control behavior.

The main conclusion is therefore not that simulation can replace physical validation, but that a well-designed digital twin can carry a substantial part of the development burden before field deployment. It can reduce risk, accelerate iteration, support structured training, and make later real-world testing more focused and more informative. For agricultural robotics, where labor scarcity, terrain variability, and crop safety are persistent constraints, this training-oriented interpretation of the digital twin offers a strong methodological direction for future work.

This study also has clear limitations. The current validation focuses primarily on self-driving behavior in a vineyard-oriented rover scenario, not yet on the full robotic pruning process. The results, therefore, support the digital twin most directly as a framework for navigation-centered development, sensor integration, and transfer preparation. The next steps should include tighter comparison between virtual and physical runs, explicit calibration of the remaining sim-to-real gaps, and expansion toward more integrated agricultural tasks in which navigation, perception, and task execution are coupled even more closely.

## Figures and Tables

**Table 1:** Methodological layers of the proposed digital-twin-based training workflow.

Layer	Main Role in the Methodology
Physical robot	Real actuators, sensors, edge computing, and field deployment.
ROS software layer	Control, perception, planning, logging, and task-level coordination.
Unity-based digital twin	Virtual robot embodiments, terrain, plants, obstacles, simulated sensors, and scene logic.
ROS–Unity bridge	Bidirectional message exchange between ROS topics/services and Unity-side interfaces.
Evaluation layer	Logging, metric computation, and transfer checks.

**Table 2:** The final observation vector.

Observation	Description	Dimension
Relative heading	Angle between rover forward vector and target vector	1
Velocity	Rover velocity on the X and Z axes	2
Target distance	Euclidean distance to the goal	1
Inclination	Rover tilt on the X and Z axes	2
LiDAR sensor	The 360 degrees are divided into 8 sections, and the closest reading is taken in each section	8
Ultrasonic sensors	5 front and 5 back facing ultrasonic sensor	10

**Table 3:** Hyperparameter list for training.

Category	Hyperparameter	Value
Architecture	Actor hidden layers	[128, 128]
	Critic hidden layers	[128, 128]
	Hidden layer activations	ReLU
	Actor output activation	Tanh
	Critic output activation	Linear (none)
	Optimizer	Adam
Optimizer	Actor learning rate	3 × 10^−5^
	Critic learning rate	3 × 10^−5^
Reinforcement Learning (RL) Parameters	Discount factor (γ)	0.99
	GAE parameter (λ)	0.95
	Entropy coefficient	0
	PPO clip range (ε)	0.2
	Value loss coefficient	0.5
	PPO epochs	8
	Mini batch size	128
	Starting standard deviation of actions taken	1
	Minimum standard deviation of actions taken	0.5
	Standard deviation decay rate	0.1
	Standard deviation decay frequency in episodes	5000
	Standard deviation decay rule	new_std^2^

**Table 4:** Summary of the test run outcomes.

Row	Method	Stuck	Collision	Success	Success Rate
Flat	Baseline	0	7	173	96.11%
Flat	RL Agent	1	0	179	99.44%
Uphill	Baseline	2	15	218	92.27%
Uphill	RL Agent	7	9	219	93.19%
Side slope	Baseline	0	21	354	94.4%
Side slope	RL Agent	1	11	363	96.8%

**Table 5:** Summary of the distances and time it took to complete in the test runs.

Row	Method	Distance in Meters (Mean ± Std)	Time in Seconds (Mean ± Std)
Flat	Baseline	1.5245 ± 0.1285	2.0543 ± 0.4665
Flat	RL Agent	1.5401 ± 0.0786	3.2284 ± 5.7791
Uphill	Baseline	1.5702 ± 0.2256	2.3997 ± 0.7983
Uphill	RL Agent	1.5952 ± 0.2871	3.2809 ± 5.4687
Side slope	Baseline	1.5737 ± 0.2386	2.4652 ± 0.7362
Side slope	RL Agent	1.5772 ± 0.2205	2.561 ± 1.5658

## Data Availability

No new data were created or analyzed in this study.
